# Eosinophilic Dermatosis of Hematologic Malignancy: Emerging Evidence for the Role of Insect Bites—A Retrospective Clinico-Pathological Study of 35 Cases

**DOI:** 10.3390/jcm13102935

**Published:** 2024-05-16

**Authors:** Andrea Michelerio, Marco Rubatto, Gabriele Roccuzzo, Marta Coscia, Pietro Quaglino, Carlo Tomasini

**Affiliations:** 1 Department of Clinical-Surgical, Diagnostic and Pediatric Sciences, University of Pavia, 27100 Pavia, Italy; 2 Dermatology Clinic, Fondazione IRCCS Policlinico San Matteo, 27100 Pavia, Italy; 3 Department of Medical Sciences, Section of Dermatology, University of Turin, 10126 Turin, Italy; 4 Department of Molecular Biotechnology and Health Sciences, University of Turin, 10126 Turin, Italy; 5 University Division of Hematology, A.O.U. Città della Salute e della Scienza di Torino, 10126 Turin, Italy

**Keywords:** eosinophilic dermatosis, insect bites, b cell chronic lymphocytic leukemia, non-Hodgkin lymphoma, eosinophils, Wells syndrome, eosinophilic cellulitis

## Abstract

**Background/Objectives:** Eosinophilic dermatosis of hematologic malignancy (EDHM) is a rare cutaneous disorder associated with various hematologic malignancies, most commonly chronic lymphocytic leukemia. Detailed clinicopathologic studies of EDHM are lacking and the pathogenesis remains enigmatic. Initially thought to be a hypersensitivity reaction to insect stings, subsequent reports have challenged this understanding. The prognostic implications of EDHM remain unclear. **Methods:** A retrospective clinicopathologic study was performed on patients diagnosed with EDHM. Hematologic and dermatologic data were reviewed. Histologic specimens were re-evaluated and lesions were classified into acute/subacute, fully developed, and chronic/regressing. **Results:** The study included 35 patients. In 80% of these patients, EDHM was diagnosed after the hematologic disorder. Approximately 45% of the cohort experienced hematologic disease progression or relapse, while 65% required therapeutic intervention during the course of their hematologic disease. In total, 15/19 CLL patients had non-mutated IgHV, a marker of a more aggressive hematologic disease course. Dermatologic lesion morphology was heterogeneous, with most lesions occurring on exposed areas, and a significant 94% of patients demonstrated lesion seasonality. Histopathologic findings were consistent with features typically associated with insect bites. In addition, examination of lesions at different chronological stages revealed substantial similarities with Wells syndrome. **Conclusions:** Our findings support the potential role of insect bites in triggering EDHM in the context of adaptive immune dysfunction. EDHM may be associated with a more aggressive disease course or may be a marker of disease progression. The observed co-occurrence of features typical of Wells syndrome in EDHM patients suggests that these conditions are part of a spectrum of disorders that vary in clinical expression.

## 1. Introduction

Eosinophilic dermatosis of hematologic malignancy (EDHM) is a cutaneous manifestation reported in several hematoproliferative and lymphoproliferative disorders, most commonly B-cell chronic lymphocytic leukemia (CLL) [1,2,3,4,5,6,7,8,9]. Clinically, it is characterized by a nonspecific pruritic eruption with pleomorphic clinical presentations ranging from papules or nodules [2,4,5,10,11,12,13,14] to blisters [1,11,12,13,15,16,17,18,19] or plaques [20]. Initially, these manifestations were considered exaggerated reactions to insect bites [10,12,17,21,22,23]. However, subsequent reports challenged this connection, as most affected individuals could not recall being bitten [2,5,13,14,24,25,26,27]. Cutaneous eruptions typically occur months to years after the diagnosis of the hematologic malignancy, although cases have been reported where the eruptions preceded the diagnosis of the underlying malignancy [2,6,13,28,29].

Histopathologically, the lesions of EDHM are characterized by a superficial and deep dense perivascular infiltrate of small lymphocytes, accompanied by numerous eosinophils, with periadnexal distribution of the infiltrate often observed [5,11,12,30,31]. However, the literature regarding dermatopathology of EDHM is scarce and comprehensive studies are lacking [2,4,5,11,12,13,14,15,16,22,27,28,29,32,33].

The aim of this study was to delineate the clinicopathologic spectrum of the disease through a review of the clinical and histopathologic features of 35 patients with EDHM.

## 2. Materials and Methods

A retrospective study was performed on 35 patients diagnosed with EDHM between 2010 and 2023 at the Dermatology Clinic of the University of Pavia and the Dermatopathology Section of the University of Turin (Italy). A total of 35 cases were retrieved and studied. In all cases, the diagnosis was based on close clinicopathologic correlation. All available dermatologic and hematologic clinical data were extracted from the medical records. These data included age at diagnosis of hematologic disease, prognostic factors, and clinical course of hematologic disease.

In addition, histopathologic specimens were re-examined by two of us (CT and AM) to ensure consistency and accuracy in the pathologic evaluation of EDHM and to verify the histopathologic features. Patients with a final diagnosis other than EDHM were excluded from the study.

The following parameters were evaluated for each case: changes in the epidermis (atrophy, acanthosis, spongiosis, basal vacuolization, exocytosis, and necrosis), dermis (edema, fibrosis, mucinosis, collagen degeneration presence of flame figures, and granulomas), and hypodermis (presence/absence of panniculitis); distribution (dermal-hypodermic, perivascular, periadnexal, lichenoid, and interstitial) and composition of the inflammatory infiltrate (lymphocytes, eosinophilic, and/or neutrophilic granulocytes, histiocytes, and mast cells); presence of leukemic lymphocytes; expression and distribution of CD30 antigen; signs of vascular damage (endothelial swollen, fibrin/thrombi, and erythrocyte extravasation); and presence of hemophagocytosis. In each sample, representative hot spots with the highest density of eosinophils in the infiltrate were identified and the number of eosinophils per high-power field (HPF; 40× objective, 400× total magnification) was calculated. The peak eosinophil count (highest number of eosinophils per HPF) was determined for each biopsy. Bullous pemphigoid was excluded by direct immunofluorescence, indirect immunofluorescence using human skin as a substrate, and enzyme-linked immunosorbent assay for the detection of autoantibodies against BP180 and BP230.

Finally, we re-evaluated the histopathologic changes in all cases based on the timing of biopsies from the onset of the lesion according to the clinical records (weeks to months). Lesions lasting one week or less were considered acute/subacute, those lasting one to three weeks were considered fully developed, and those lasting more than three weeks were considered chronic/regressing.

## 3. Results

### 3.1. Clinical Results

A total of 35 patients were enrolled—16 males, age range from 54 to 82 (mean age, 72; median age, 72), and 19 females, age range from 53 to 87 (mean age, 68; median age, 66) at the time of diagnosis of hematologic disease.

The cohort included a variety of hematologic malignancies: 27 patients had CLL (77%), 2 had B-cell monoclonal lymphocytosis (cases #18 and #20), 2 had follicular lymphoma (cases #9 and #11), 1 had mantle cell lymphoma (case #23), 1 had multiple myeloma (case #19), 1 had marginal zone lymphoma (case #10), and 1 had chronic myelomonocytic leukemia (case #29).

In 28 patients (80%), the diagnosis of EDHM followed the diagnosis of hematologic disease. In five patients (14%) (#7, #8, #12, #20, and #31), skin lesions preceded the hematologic diagnosis. Specifically, the onset of dermatologic symptoms occurred 18 months before the hematologic diagnosis in patient #7, 12 months before in both patients #8 and #12, 3 years before in patient #20, and 17 years before in patient #31. In two cases (#33 and #34) the diagnosis was concomitant.

**Figure 1:**
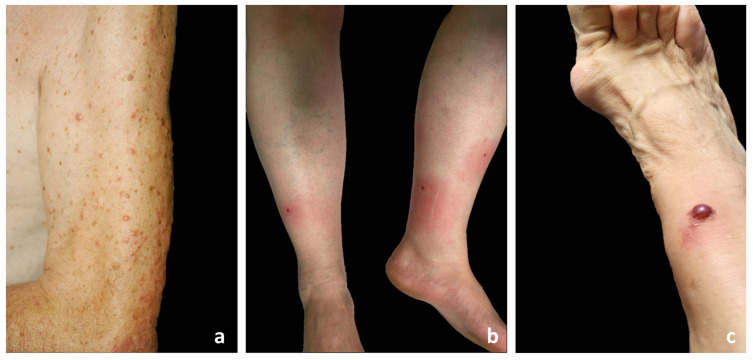
EDHM lesions. (**a**) Diffuse erythematous papules confluent in plaques on the upper extremities; (**b**) Hypodermitis-like erythematous plaques centered by a crust on the lower limbs; (**c**) Bullous lesion with hemorrhagic and serous content on an erythematous plaque on the lower limb.

**Figure 2:**
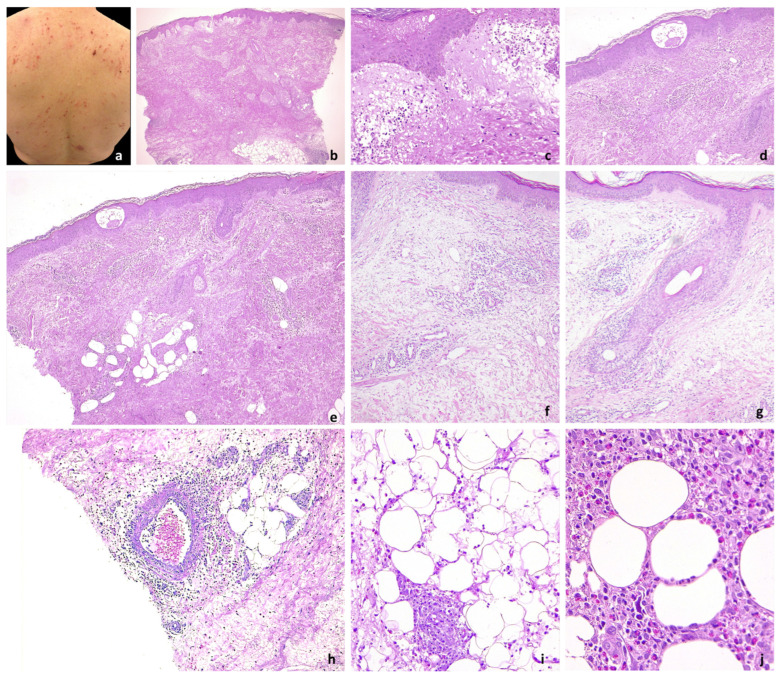
Acute/subacute EDHM lesions. (**a**) Numerous and widespread acute/subacute lesions on the trunk. (**b**) A superficial and deep perivascular, periadnexal, and interstitial infiltrate with numerous eosinophils in the papillary and mid-dermis extending into the subcutaneous tissue. (**c**,**d**) Epidermal spongiosis, papillary dermal edema, and eosinophilic epidermal vesicles are seen. (**e**) The infiltrate involves adnexal structures, both eccrine (**f**) and follicular (**g**). (**h**) The inflammatory reaction also involves the hypodermis, with vasculitis of medium-sized vessels, lobular and septal eosinophilic panniculitis (**i**), and eosinophilic rim of subcutaneous fat lobules (**j**).

**Figure 3:**
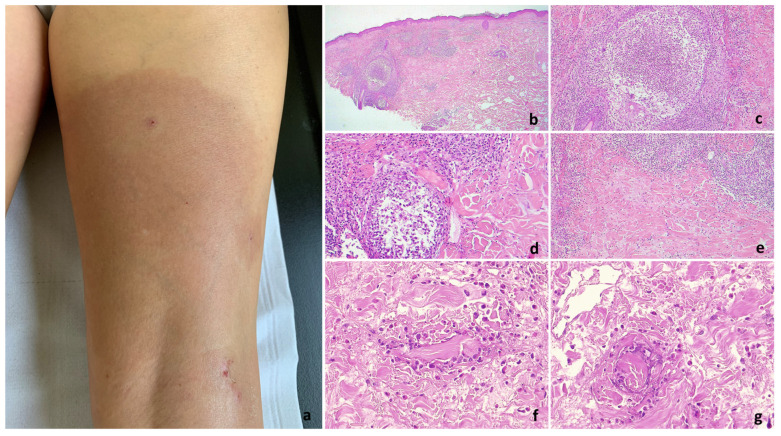
Fully developed EDHM lesion. (**a**) An extended erythematous plaque involving the right leg. (**b**,**c**) Eosinophilic folliculitis associated with significant perifollicular and dermal collagen sclerosis (**d**). (**e**) Extensive collagen sclerosis is present in deeper dermal layers. (**f**,**g**) Eosinophils around degenerated collagen bundles forming flame figures of eosinophilic granulomas.

**Figure 4:**
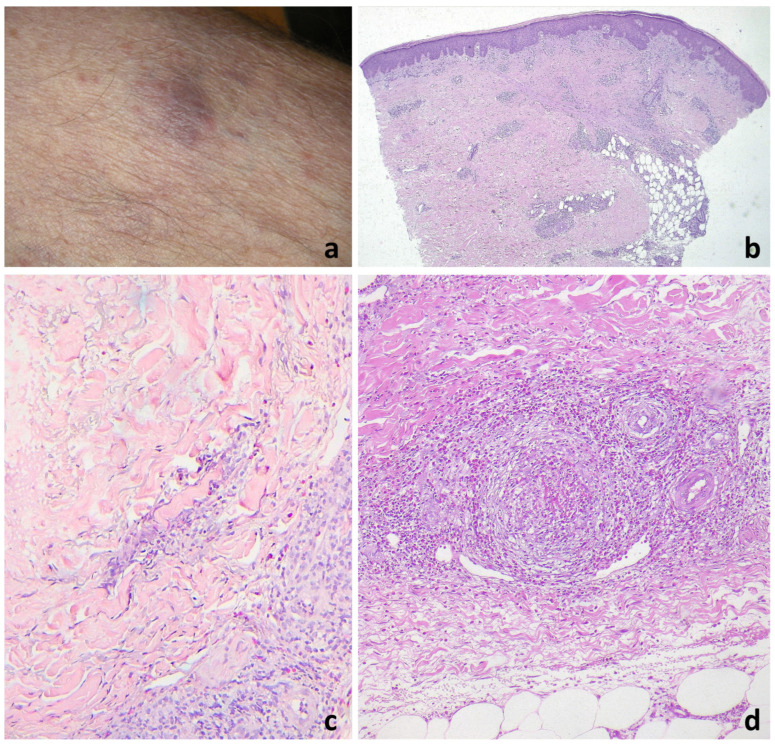
Fully developed EDHM lesion. (**a**) An erythematous-violaceous nodule on the leg. (**b**) The infiltration is much less extensive than in the acute phases whilst collagen bundles are thickened. (**c**) Flames and eosinophilic granulomas are still visible. (**d**) Vasculitis is seen in the deeper dermis.

**Figure 5:**
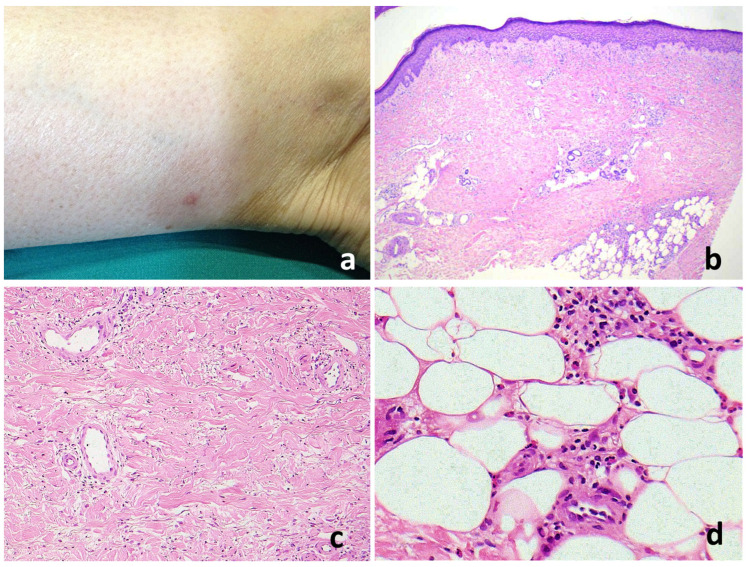
A late-stage/regressing EDHM lesion. (**a**) A nuanced erythematous plaque on the leg. (**b**) Sparse inflammatory infiltrate and prominent dermal sclerosis reminiscent of morphea. The epidermis is normal but the dermal–epidermal junction is flattened due to attenuated rete ridges. Dermal edema is absent. There is a marked reduction in capillaries and small vessels, which are replaced by densely packed collagen bundles. (**c**) The eccrine glands are atrophic and encapsulated within the thickened dermis, while the underlying dermis appears homogenized and hyalinized. (**d**) Eosinophilic infiltration may still be seen in the subcutaneous tissue around the fat lobules.

The seasonality of lesions was observed in 33/35 patients: 14 developed lesions specifically in the summer months, 16 in the spring, and 3 in the autumn. The lesion morphology was variably described as erythematous papules, nodules, and plaques (Figure 1a,b, Figure 2a, Figure 3a, Figure 4a, and Figure 5a) and blisters were reported in 7 patients (Figure 1c) (#3, #10, #15, #18, #26, #33, and #34). Dermatologic lesions predominantly involved the limbs in 27 patients and were diffuse in 8 patients (cases #5, #8, #12, #26, #27, #29, #31, #35).

Information on the hematologic course of the disease was available for 29 patients. Disease progression or relapse was observed in 16 patients (cases #4, #5, #6, #7, 8, #10, 13, #14, #15, #17, #21, #22, #29, #31, #32, and #33), while 9 patients had stable disease (cases #2, #12, #19, #24, #25, #26, #30, #34, and #35) and 4 patients had complete remission (cases #1, #3, #9, and #11) at the last visit. Median survival was 8 years.

Twenty-four patients required therapeutic intervention during the course of their hematologic disease. Among the 27 CLL patients, the mutation status of the immunoglobulin heavy chain (IgHV) gene was determined in 19 patients, of whom 15 were found to be unmutated (78%). In 22 CLL patients, the CLL FISH panel for the detection of chromosome aberrations useful for prognosis and time to treatment was used: no alterations were found in 5 patients, whilst 7 patients had trisomy 12, 3 patients had 17q deletions, 3 patients had 13q deletions, 1 patient had 13q and 11q deletions, and 1 patient had 13q and 17p deletions. Wild-type p53 function was assessed in 13 patients, with loss of function in 3 patients. The clinical data of our patients are shown in Table 1.

### 3.2. Histopathologic Results

Histopathologic examination revealed epidermal acanthosis (21/35) and mild spongiosis (21/35), up to formation of eosinophilic intraepidermal vesicles (9/35) (Figure 2b–e, Figure 4b). Papillary dermis edema was observed in 11 cases and subepidermal blistering in 7 cases (Figure 2b,c).

Superficial and deep perivascular (35/35), interstitial (23/35), and periadnexal (29/35) infiltrates with eosinophils and lymphocytic elements were observed in the papillary and mid-dermis (Figure 2 and Figure 3). The periadnexal infiltrate involved the hair follicle in 13/35 patients, up to eosinophilic folliculitis with sebaceous gland involvement (Figure 2e–g and Figure 3b,c). The infiltrate was wedge-shaped in six cases and nodular in one case. Eosinophils per HPF ranged from 25 to 300 (mean 85). The infiltrate was eosinophilic-rich in 22/35 cases and lymphocytic-rich in 9 cases. Interstitial edema was common (21/35). The reticular dermis was characterized by variable sclerosis with piecemeal fragmentation of collagen in all cases (Figure 2b,e, Figure 3b–e and Figure 4b,c). Eosinophils surrounding collagen bundles and forming small granulomas (9/35) and flame figures (14/35) were observed (Figure 3f,g and Figure 4c).

The inflammatory reaction in 24/35 also involved the hypodermis, with lobular and septal eosinophilic panniculitis (Figure 2b,h,i) and eosinophilic rimming of subcutaneous fat lobules (7/35) (Figure 2j). Vasculitis of medium-sized vessels was also observed in 14/35 patients (Figure 2h and Figure 4d). Four cases showed a small leukemic B-cell component (CD20−/+, CD79a+, and CD23+), not exceeding 10% of the infiltrate.

Regarding the histopathologic life of lesions, the majority of cases were identified as acute/subacute (26), followed by fully developed (5) and late stage/regressing (4). Notably, granulomas and flame figures were present only in fully developed and late-stage lesions. In addition, the extent of the infiltrate decreased progressively from acute/subacute lesions to late-stage/regressing lesions, while the degree of sclerosis increased. In late-stage lesions, the infiltrate was sparse, with few eosinophils and diffuse sclerosis (Figure 5).

Direct immunofluorescence was performed in 24 patients and the results were heterogeneous and non-specific.

## 4. Discussion

The pathogenesis of EDHM remains unclear, making patient management challenging. The present study provides insight into the clinical and histopathologic features of this disease, confirming established concepts and highlighting new findings. In our study, the majority of patients (77.1%) had CLL, which is consistent with data in the literature. The higher incidence of CLL compared to other hematologic malignancies may be explained by the dysregulation of the T cell compartment, which is characterized by an imbalance favoring a Th2 over Th1 response [35,36]. Polarization of the Th2 response may lead to IL-4 and IL-5 production and eosinophil recruitment. The role of insect bites in the pathogenesis of EDHM is controversial. The main criticism of those who deny the role of insect bites is the lack of recall of insect bites in most patients and the failure of preventive measures [2,5,13,14,24,25,26,27]. Nevertheless, in our study, the seasonal pattern and lesion topography supports the role of insect bites [34,37]. In fact, most of the lesions occurred on exposed areas such as the limbs, while only eight cases (22%) involved the trunk [34]. In addition, although mosquitoes are often acknowledged and investigated as the typical offender, it should be noted that there are other offenders and some, especially mites, are often difficult to identify and the patient may not feel the bite or notice the insect when it bites [38,39,40]. For example, *Pyemotes ventricosus* dermatitis is characterized by a delayed reaction (10–28 h) to the bites, with no initial prickle when bitten, manifesting with urticarial papules surmounted by punctate vesicles, often centered by a hemorrhagic spot [41]. This condition also has a seasonal prevalence, occurring primarily between May and November. The distribution of the rash depends mainly on the circumstances of the contact and may include the neck and arms, as well as areas covered by occlusive clothing such as the shoulders and upper torso. The patient is often reluctant to accept the diagnosis of insect bites, so a definitive diagnosis may be made only by clinical features, patient history, or mite identification [41]. Other potential elusive offending agents include Trombiculidae larvae, particularly *Neotrombicula autumnalis*, and *Cheyletiella* spp., which is commonly associated with pet infestations [42]. In particular, *Neotrombicula autumnalis* predominates during the late summer and fall seasons. This seasonal pattern may explain the increase in lesion cases in the fall, a time when mosquitoes are typically less abundant in our latitudes [42]. The use of advanced observational techniques, such as Fluorescence–Advanced Videodermatoscopy, in diagnostic procedures has the potential to accurately identify difficult-to-see etiologic agents, including mites [40]. These advances are not only diagnostic but potentially preventative, as they could contribute to a more targeted approach to patients with parasitic infestations.

Furthermore, the failure of preventive measures may also be due to the reactivation of previous lesions caused by new insect bites [43]. It is important to note that eosinophils, unlike neutrophils, can survive in the tissue for weeks, depending on the cytokines that are released [42]. In EDHM, the persistence of eosinophils in lesional tissue may further contribute to the recurrent course and clinical and pathologic findings of the disease.

The histopathologic observations of our study are consistent with those documented in the literature on insect bites and parasitic infestations. Characteristic histologic features of insect bites/parasitic infestations include an inflammatory infiltrate in the dermis, both superficial and deep, surrounding blood vessels, adnexal structures, and interspersed within the tissue, consisting primarily of lymphocytes and eosinophils, with frequent observation of spongiosis in the overlying epidermis. Sometimes, the infiltrate evolves into intraepidermal vesicles or even progresses to epidermal necrosis. Flame figures and vasculitis can be observed [44,45]. In our study, 31/35 of the skin biopsies showed prominent periadnexal involvement (either sweat glands or hair follicles). Ackerman et al. suggested that the periadnexal and especially the perifollicular distribution of the infiltration may be due to the fact that the insect is attracted to microorganisms and lipids present in the follicular ostia and sebaceous glands [44]. Wedge-shaped infiltrates and acrosyringial involvement are also consistent with arthropod and insect bites due to their attraction to sweat, as well as flame figures and vasculitis [45,46].

The prognostic significance of EDHM in hematologic malignancies remains controversial. Typically, CLL has a variable clinical course and may not require treatment in all patients. However, in our cohort, a significant number of patients required therapeutic intervention for progressive or symptomatic hematologic disease [47], suggesting that EDHM may potentially represent a worsening clinical sign of the underlying hematologic malignancy. This observation is consistent with documented trends in the literature [2,4,13].

The mutational status of the variable region of the immunoglobulin heavy chain (IgHV) gene is also an important prognostic marker [48]. The *IGH* genes are responsible for the production of immunoglobulin heavy-chain proteins and play a critical role in antigen recognition by the immune system. By altering the genes encoding the IgHV region in B cells, somatic hypermutation increases the efficiency of the immune response in the lymph nodes. B-cell malignant transformation can occur either before or after this process and therefore CLL has two types of B-cell clones, mutated and unmutated, according to their mutational status of the *IgHV* gene [49]. Mutations in the IgHV gene are associated with a less aggressive form of CLL, possibly because they result in reduced immunoglobulin autoreactivity, which affects the severity of the disease by reducing the ability of the B-cell receptor to support the survival of leukemic cells [49,50,51]. Conversely, the absence of an IgHV mutation has been associated with an unfavorable prognosis and as a predictor of shorter progression-free survival [49,50,51,52]. Notably, in our study, 15/19 CLL patients had an unmutated IgHV mutation status.

Our study includes two patients with monoclonal B-cell lymphocytosis. Monoclonal B lymphocytosis is an asymptomatic condition in which individuals have a persistent elevation of the clonal B-cell population in the peripheral blood lymphocytes that does not reach the level at which it would be considered CLL (≥5 × 10^9^ B cells/L) without other features of a B-cell lymphoproliferative disorder [53,54]. To our knowledge, the association between EDHM and monoclonal B-cell lymphocytosis has not been reported in the literature. Therefore, whether or not EDHM in patients with MBL is predictive of conversion to overt CLL requires additional cases and longer follow-up.

The clinical and histopathological findings of our series share many similarities with what is described under the term Wells syndrome (WS). First described in 1971 by G.C. Wells as “recurrent granulomatous dermatitis with eosinophilia” [55], WS is a rare dermatosis without ethnic or gender predilection that primarily affects adults [56]. The etiology is unknown, although some cases have been associated with various underlying conditions and triggers, including hematologic disorders [20,57,58,59,60,61,62,63,64,65], hypersensitivity reaction to insect bites [40,66,67], medications [68,69,70], infections [71,72,73], and vaccinations [56,74,75,76]. Peripheral eosinophilia, leukocytosis, or elevated inflammatory markers may be observed [58,77]. Systemic symptoms such as arthralgia and fever may occur in some cases [55,70]. Clinical manifestations are often preceded by prodromal pruritus or pain and are polymorphic, including edematous nodules and plaques resembling cellulitis [44,69,78]. Not infrequently, lesions may become bullous, resembling bullous pemphigoid [79,80]. The extremities are predominantly involved [78,81]. The lesions rapidly evolve into plaques that resolve spontaneously over 2–8 weeks without scarring [69,70,82]. Multiple recurrences are typically observed, with variable locations and time intervals from the previous flare [78].

All of these clinical characteristics mirror those of our patients. Furthermore, bites or stings from arthropods such as mosquitoes, fleas, honeybees, ticks, spiders, and even centipedes have been reported as potential triggers of WS [38,40,59,66,67,83]. Similarly, hematologic malignancies have been mentioned as potential initiators of WS [20,56,57,58,59,60,61,62,63,64,65,84].

Whilst WS is a prototypical eosinophilic dermatosis, the histopathologic features are dynamic depending on the time of biopsy [85,86]. In the acute/subacute stage, a variably dense perivascular, periadnexal, and interstitial inflammatory infiltrate composed of eosinophils mixed with lymphocytes and macrophages fills the dermis, often extending into the subcutaneous fat. Dermal edema may be prominent, leading to subepidermal bulla, while epidermal spongiosis may lead to vesicle formation. Over time, basophilic degeneration and/or piecemeal fragmentation of collagen with the formation of small granulomas supervene. Typically, degranulation of eosinophils with deposition of eosinophil basic protein on collagen bundles leads to the formation of flame patterns which, although characteristic, are not specific for the disease. In fact, they are seen in many other unrelated dermatoses in which eosinophils are abundant, such as insect bites, bullous pemphigoid, eczema, prurigo, scabies, herpes gestationis, Churg-Strauss syndrome, and parasitic infections [56,86]. Vasculitis is usually absent [59,69].

In our study, histopathologic features of skin biopsies correlated with lesion duration at the time of biopsy, similar to WS syndrome lesions, further highlighting the clinicopathologic similarities between the two diseases. Indeed, the corresponding figures illustrate the dynamic nature of the disease: acute/subacute lesions are characterized by epidermal spongiosis and severely edematous papillary dermis, along with a superficial and deep eosinophilic dermal infiltrate. Eosinophilic panniculitis suggests the involvement of deeper skin layers early in the disease.

As the disease progresses, dermal edema and inflammation decrease whilst sclerosis supervenes. Between the collagen bundles, eosinophils and histiocytes are found in the interstitial spaces, forming flame patterns and small eosinophilic granulomas around the deteriorating collagen bundles, indicating ongoing inflammation and tissue remodeling.

At this evolutionary stage, the interstitial and palisaded granulomatous dermatitis present differential diagnoses with other granulomatous diseases such as annular elastolytic giant cell granuloma (AEGCG) [87] and reactive granulomatous dermatitis (RGD) [88,89].

AEGCG is an idiopathic granulomatous dermatosis characterized by plaques with a raised erythematous border and central atrophy, typically found on sun-exposed areas [87]. RGD is a unifying term to describe overlapping entities with varying clinical presentations but which histologically share the common thread of granulomatous inflammation [88]. In particular, RGD may be associated with other conditions, including autoimmune disorders, hematologic malignancies, drug reactions, and infectious diseases [88,89].

In the chronic or regressing phase, the inflammatory infiltrate diminishes significantly and dermal sclerosis becomes the predominant feature. The skin structure shows extensive collagen deposition, resulting in a dense dermal layer with reduced vascularity. This stage warrants differential diagnosis, especially with morphea and eosinophilic fasciitis (EF) [90]. The latter is a rare connective tissue disorder that predominantly affects the limbs with progressive induration and thickening of the skin and soft tissues, variable eosinophilic infiltration, peripheral eosinophilia, hypergammaglobulinemia, and an elevated erythrocyte sedimentation rate [91]. Hematologic disorders coexist in less than 10% of cases of EF, suggesting a close relationship between these conditions within a broader spectrum of eosinophilic dermatoses [91,92].

In conclusion, our study supports the role of insect bites in triggering EDHM in the context of adaptive immune dysfunction. Our hypothesis is also that EDHM may have a negative prognostic value and in some cases may be a hallmark of disease progression. Therefore, closer follow-up may be indicated. The co-occurrence of WS features in EDHM patients supports the idea of a spectrum of disorders with different nuances in clinical expression.

## Figures and Tables

**Table 1:** Clinical data of study patients.

Case/ Gender	Hematologic Disease	IgHV Mutational Status	FISH Panel LLC	TP53	Disease Relapse/ Progression, Follow-Up	Age at Diagnosis of Hematologic Disease (y)	Timing of EDHM Onset to Hematologic Diagnosis/Chemotherapy	Lesion Topography	EDHM Clinical Presentation	Survival (Years)
1/F *	SLL/CLL	mutated	trisomy 12	wt	CR, LFU	77	One year after chemotherapy	upper and lower extremities, face	Polymorphic; purpuric papules and nodules on the limbs, urticarial plaques on the face	8
2/F *	CLL	unmutated	del(11q); del(13q)	wt	SD, death	76	3 months after chemotherapy	upper and lower extremities, face	Monomorphic; urticarial plaques centrally excoriated	10
3/F *	CLL	unmutated	del(13q)	wt	CR, alive	87	3 years after chemotherapy	lower extremities, face	Polymorphic; blisters on the limbs, urticarial plaques on the face	8
4/M *	CLL	unmutated	no alterations	wt	P, LFU	57	18 months after chemotherapy	lower extremities	Monomorphic; purpuric urticarial plaques centrally excoriated	8
5/F *	CLL	unmutated	no alterations	wt	P, death	76	One year after chemotherapy	upper and lower extremities, face, trunk	Polymorphic; purpuric papules and nodules on the limbs, urticarial plaques on the face and trunk	7
6/F *	CLL	mutated	del(13q)	mut	P, alive	77	16 years after the first chemotherapy	upper extremities	Monomorphic; urticarial plaques	14
7/F *	CLL	unmutated	no alterations	mut	P, LFU	61	18 months before hematological disease diagnosis	upper and lower extremities, face	Polymorphic; panniculitis-like plaques, excoriated erythematous papules, urticarial plaques	4
8/F *	CLL	unmutated	trisomy 12	wt	P, alive	66	One year before hematological disease diagnosis	upper and lower extremities, trunk	Monomorphic; centrally excoriated erythematous papules and nodules	6
9/M *	FL	NA	NA	NA	RC, alive	71	One year after diagnosis and chemotherapy	upper and lower extremities	Monomorphic; centrally excoriated erythematous papules and nodules	7
10/F *	MZL	NA	NA	NA	P, alive	66	During chemotherapy (third cycle)	upper and lower extremities	Polymorphic; urticarial plaques on the upper extremities, blisters on the lower extremities	5
11/F *	FL	NA	NA	NA	RC, alive	78	After fourth chemotherapy cycle	upper and lower extremities	Polymorphic; erythematous papules, cellulitis-like plaques on the upper extremities	5
12/M *	CLL	unmutated	no alterations	wt	SD, death	68	One year before hematological disease diagnosis	upper and lower extremities, face, trunk	Polymorphic; panniculitis-like plaques; urticarial plaques, centrally excoriated erythematous papules	5
13/M *	CLL	unmutated	del(13q), del(17p)	mut	P, LFU	65	Six years after chemotherapy	upper and lower extremities	Monomorphic; centrally excoriated erythematous papules and nodules	11
14/F *	CLL	unmutated	del(13q)	wt	P, alive	53	Concomitant with first chemotherapy cycle	lower extremities	Monomorphic; centrally excoriated urticarial plaques	10
15/M *	SLL/CLL	unmutated	no alterations	NA	P, LFU	67	Three years after diagnosis and chemotherapy	upper and lower extremities	Monomorphic; excoriated vesico-papules	1
**16/M**	CLL	/	/	/	/	82	After the hematologic disease diagnosis	upper and lower limbs	erythematous papules and nodules	NA
**17/M**	CLL	unmutated	del17q	/	P, death	71	8 months after the hematologic disease diagnosis	lower limbs	erythematous papules and plaques	17
**18/M**	monoclonal B cell lymphocytosis	/	/	/	/	79	After the hematologic disease diagnosis	upper and lower limbs	erythematous urticarial plaques and blisters	NA
**19/F**	multiple myeloma	/	negative	/	SD, death	56	After the hematologic disease diagnosis	lower limbs	erythematous papules and urticarial plaques	7
**20/M**	monoclonal B cell lymphocytosis	/	/	/	/	78	36 months before the hematologic disease diagnosis	lower limbs	Erythematous-violaceous nodules	NA
**21/F**	CLL	mutated	del17q	/	P, death	58	18 years after the hematologic disease diagnosis	lower limbs	erythematous urticarial plaques and erythematous nodules	25
**22/M**	CLL	unmutated	tris12	/	P, death	71	7 months after the hematologic disease diagnosis	upper limbs	erythematous nodules	3
**23/M**	mantle cell lymphoma	/	/	/	/	81	After the hematologic disease diagnosis	upper limbs	excoriated erythematous papules	NA
**24/F**	CLL	/	/	/	SD, alive	63	After the hematologic disease diagnosis	upper limbs	erythematous papules and urticarial plaques	11
**25/M**	CLL	unmutated	negative	wt	SD, death	74	3 months after the hematologic disease diagnosis	upper and lower limbs	excoriated erythematous papules	3
**26/F**	CLL	unmutated	tris12	/	SD, death	70	3 months after the hematologic disease diagnosis	diffuse	erythematous urticarial plaques and blisters	9
**27/M**	CLL	/	/	/	/	73	After the hematologic disease diagnosis	diffuse	erythematous papules	NA
**28/F**	CLL	/	tris12	/	/	72	45 months after the hematologic disease diagnosis	lower limbs	erythematous papules	NA
**29/M**	CMML	/	negative	/	P, death	76	14 months after the hematologic disease diagnosis	diffuse	excoriated erythematous papules	2
**30/M**	CLL	/	/	/	SD, death	80	36 months after the hematologic disease diagnosis	upper and lower limbs, face	monomorphic erythematous papules and urticarial plaques	11
**31/M**	CLL	unmutated	tris12	/	P, alive	54	17 years before the hematologic disease diagnosis	diffuse	erythematous nodules	14
**32/F**	CLL	/	del17q	/	P, alive	55	After the hematologic disease diagnosis	upper and lower limbs	erythematous nodules and urticarial plaques	7
**33/F**	CLL	/	/	/	P, alive	86	Concomitant the hematologic disease diagnosis	upper and lower limbs	erythematous nodules and blisters	3
**34/F**	CLL	/	/	/	SD, alive	61	Concomitant the hematologic disease diagnosis	upper and lower extremities	hemorrhagic plaques and nodules, blisters	1
**35/F**	CLL	unmutated	tris12	wt	SD, alive	64	2 years after the hematologic disease diagnosis	diffuse	erythematous plaques	2

* Cases from [34]. M, male; F, female; SLL, small lymphocyte lymphoma; CLL, chronic lymphocytic leukemia; FL, follicular lymphoma; MZL, marginal zone lymphoma; CMML, chronic myelomonocytic leukaemia; IgHV, immunoglobulin heavy chain gene; wt, wild type; LFU, lost at follow-up; P, progression; SD, stable disease.

## Data Availability

The original contributions presented in the study are included in the article, further inquiries can be directed to the corresponding authors.
